# The genetic architecture of constitutive and induced trichome density in two new recombinant inbred line populations of *Arabidopsis thaliana*: phenotypic plasticity, epistasis, and bidirectional leaf damage response

**DOI:** 10.1186/1471-2229-14-119

**Published:** 2014-05-05

**Authors:** Rebecca H Bloomer, Alan M Lloyd, V Vaughan Symonds

**Affiliations:** 1 Institute of Fundamental Sciences, Massey University, Private Bag 11222, Palmerston North 4442, New Zealand; 2 Institute for Cellular and Molecular Biology, University of Texas, Austin, TX, USA

**Keywords:** *Arabidopsis*, Trichome density, QTL, Plant defense, Genetic architecture, Natural variation

## Abstract

**Background:**

Herbivory imposes an important selective pressure on plants. In *Arabidopsis thaliana* leaf trichomes provide a key defense against insect herbivory; however, trichome production incurs a fitness cost in the absence of herbivory. Previous work on *A. thaliana* has shown an increase in trichome density in response to leaf damage, suggesting a mechanism by which the cost associated with constitutively high trichome density might be mitigated; however, the genetic basis of trichome density induction has not been studied.

**Results:**

Here, we describe the mapping of quantitative trait loci (QTL) for constitutive and damage induced trichome density in two new recombinant inbred line populations of *A. thaliana*; mapping for constitutive and induced trichome density also allowed for the investigation of damage response (plasticity) QTL. Both novel and previously identified QTL for constitutive trichome density and the first QTL for induced trichome density and response are identified. Interestingly, two of the four parental accessions and multiple RILs in each population exhibited lower trichome density following leaf damage, a response not previously described in *A. thaliana*. Importantly, a single QTL was mapped for the response phenotype and allelic variation at this locus appears to determine response trajectory in RILs. The data also show that epistatic interactions are a significant component of the genetic architecture of trichome density.

**Conclusions:**

Together, our results provide further insights into the genetic architecture of constitutive trichome density and new insights into induced trichome density in *A. thaliana* specifically and to our understanding of the genetic underpinnings of natural variation generally.

## Background

Insect herbivory is a significant selective pressure in plant populations, with herbivores consuming some 10-15% of all plant biomass produced annually [1]. In response, plants produce an array of deterrents, ranging from physical structures such as thorns or trichomes to a variety of unpalatable or toxic chemical defenses. The model plant species *Arabidopsis thaliana* employs both physical and chemical defense strategies: most natural accessions produce both leaf trichomes and glucosinolates, a group of defensive secondary metabolites produced by members of the Brassicales. In natural populations of *A. thaliana* and in the closely related *A. lyrata*, leaf trichomes provide protection against insect herbivory [2,3]. Damage resulting from herbivory is negatively correlated with trichome density [3], with predation in the field shown to exert positive selection on increased trichome density [4]. However, trichome production also has fitness costs in *A. thaliana*, both in terms of fruit production [3] and standardized growth rate [5]. Similarly, a fitness cost for trichomes has been shown in the wild relatives *A. kamchatica*[6] and *A. halleri* ssp *gemmifera*[7], with evidence of divergent selection for trichome density identified in *A. kamchatica* and *A. lyrata*[8]. Reflecting these conflicting selection pressures, constitutive trichome density is highly variable among natural accessions of *A. thaliana* with a strong genetic basis to the observed variation under controlled conditions [9-11].

Constitutive defense mechanisms are typically assumed to be costly, diverting resources away from growth and reproduction; in contrast, induced defense responses allow plants to avoid high-level defensive investments unless required. Although induction of trichome initiation has not been demonstrated in the field in *A. thaliana*[3], trichome production is induced by artificial wounding of early leaves [12]. Such phenotypic plasticity implies a mechanism by which *A. thaliana* may offset some of the cost of producing trichomes, investing in higher density only when required. Previous QTL mapping studies have investigated the genetic architecture of constitutive trichome density in *A. thaliana*[9,11,13-15]. However, the genetic basis of induced trichome density and plasticity of trichome density have not been studied, although these are perhaps more meaningful traits in nature, as they capture the ability of plants to respond to the dynamic selective forces at play.

The molecular genetic basis of trichome initiation on *A. thaliana* leaves is relatively well understood. Initiation of trichomes on the leaf lamina requires interaction between the WD repeat protein TRANSPARENT TESTA GLABRA (TTG1), one of the functionally overlapping bHLH proteins GLABRA3 (GL3) or ENHANCER OF GL3 (EGL3) [16], and the trait-specific R2R3 MYB GLABRA1 (GL1) [17], forming a complex that activates downstream genes involved in trichome initiation. A suite of R3 MYBs act as suppressors of initiation in surrounding cells, generating a spacing pattern across the leaf [18]. Initiation at the leaf margin is similarly controlled, with GL3 or TT8 [19] interacting with TTG1 and MYB23 to activate downstream genes. Phytohormones also play a role in regulating trichome density on rosette leaves and inflorescence organs [20-22]; *GL1* and *GL3* expression are induced by gibberellins [19,23], with the DELLA family of repressors playing a role in this signalling [22]. *GL3* is up-regulated by both exogenous [12,19] and endogenous jasmonic acid [24] via interaction with JAZ proteins [25], linking induction of trichome initiation following wounding to the TTG1 pathway. Previous QTL and association mapping studies have suggested TTG1 pathway genes as good candidates for trichome density variation [9,13,26], and recent studies have shown that natural variation in the R3 MYB repressor *ETC2*[26], the bHLH *ATMYC1*[27], and the R2R3 MYB *GL1*[10] underlies quantitative variation for trichome density.

Quantitative trait locus (QTL) and genome wide association mapping approaches are key, complementary approaches in characterizing genetic architecture and identifying candidate genes underlying natural phenotypic variation [28]. Genome-wide association studies (GWAS) provide high resolution of mapped loci and a wide sampling of genetic variation, but can be confounded by false positive or negative associations due to population structure or overcorrection for population structure, and may fail to uncover rare allele effects [29,30]. Mapping in Recombinant Inbred Line (RIL) populations typically has lower resolution than GWAS but resolves population structure and rare allele effects (assuming the alleles are present in the parents). The use of both GWAS and experimental populations such as RILs together can significantly improve the identification of candidate genes [31]. Thus, the development of experimental populations which incorporate new genetic variation remains an important objective. Here, we describe QTL mapping results from two new *A. thaliana* RIL mapping populations, Hi-0 x Ob-0 (HO) and St-0 × Sf-2 (SS). The parental accessions were chosen based on variation in several phenotypes to create populations which would be broadly useful to the *Arabidopsis* research community; to our knowledge, these are the first publically available RIL mapping populations to include these four accessions.

The new RIL populations are used here to examine the genetic architecture of constitutive and induced trichome density on early leaves, and to assess the genetic basis of the response of plants to damage. Although constitutive trichome density has been mapped previously [9,11,13-15], mapping in these new populations affords unique comparative analyses, given the trichome density phenotypes of the parent accessions; further, previous studies have not investigated induced changes in trichome density resulting from variable environments or herbivore-like damage. This research seeks to address several questions: 1) How genetically independent are constitutive and induced trichome density? 2) How variable is the trichome density response to leaf damage? 3) Is there a genetic basis to variation in trichome density plasticity? 4) To what extent do epistatic interactions underlie trichome density variation?

## Results

### RIL population genotyping and linkage map construction for Hi-0 x Ob-0 and St-0 x Sf-2

Hi-0 x Ob-0 (HO) was genotyped with 55 markers (8–14 markers per chromosome), while St-0 × Sf-2 (SS) was genotyped with 67 markers (9–16 markers per chromosome; Additional file 1). Residual heterozygosity across all markers was 1.12% in the HO population and 1.36% in the SS population (Table 1). This is low and similar to that reported for other RIL populations [15,32,33] but slightly higher than the <1% expected beyond the F_7_ generation, which may reflect the conservative approach taken to allele calling (described in Methods) or heterozygote advantage. Some degree of segregation distortion (SD) was observed in both populations (Additional file 2). HO RILs exhibited segregation distortion localized to regions of chromosomes I, IV and V with preferred parental alleles varying by genomic region. In the SS population, St-0 accounted for 60.7% of alleles observed across all markers primarily as a result of strong distortion favoring St-0 alleles across all of chromosome I, the majority of chromosome V and on localized regions of chromosome II. Localized SD is commonly observed in *A. thaliana* RIL populations and typically attributed to unintentional selection during RIL development, for example, for traits affecting germination or flowering [32,33]. However, SD between loci on chromosomes I and V biased toward retention of the same parental allele at both loci has been observed in a number of mapping populations involving a range of accessions (e.g., [32,33] and there is some evidence that this may be due to genomic incompatibilities [34].

**Table 1:** RIL population details

**Population**	**Number of RILs genotyped**	**Number of markers**	**Total map length (cM)**	**Average marker distance (cM)**	**Residual heterozygosity (%)**
Hi-0 x Ob-0	181*	55	479	9.60	1.12
St-0 x Sf-2	181*	67	478	7.72	1.36

*seven RILs were removed from each population due to low genotype success.

**Figure 1:**
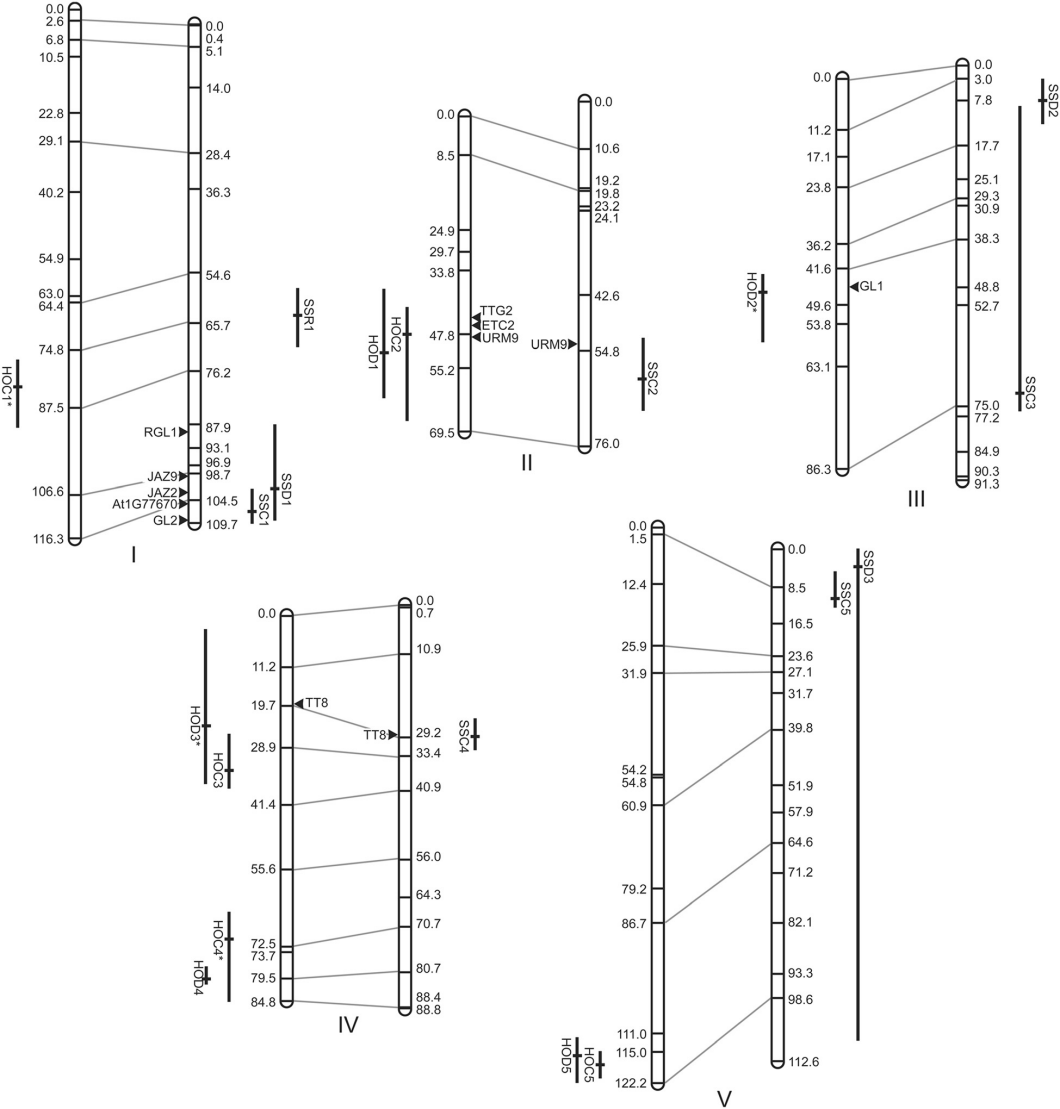
**Linkage maps and mapped QTL.** Aligned linkage maps for the five *A. thaliana* chromosomes for the Hi-0 x Ob-0 (left) and St-0 x Sf-2 (right) RIL mapping populations, with marker positions shown in cM. The peak LOD positions for QTL identified for each of the three traits are indicated by short solid black horizontal bars; Bayes’ credible intervals are indicated by perpendicular bars. Interacting QTL are indicated with an *. QTL are labelled by population and trait as in Table 3: HOC = Hi-0 x Ob-0 Constitutive; HOD = Hi-0 x Ob-0 Damage induced; SSC = St-0 x Sf-2 Constitutive; SSD = St-0 x Sf-2 Damage induced; SSR = St-0 x Sf-2 Response to leaf damage. Positions and names of candidate genes are marked with a black triangle.

Marker order on linkage maps was consistent with physical position for most markers, with the exception of several tightly linked marker pairs in each population and three markers around the centromere of chromosome V in HO (Additional file 1). These markers were constrained to match their order on the physical map during linkage map construction in joinmap[35]; the likelihood of the constrained versus unconstrained marker orders was tested in R/qtl [36], showing only nominally less strong support for the constrained marker order (markers indicated in Additional file 1). Although marker order may vary among natural accessions of *A. thaliana*, we have conservatively constrained the order here and found that it has no effect on mapping results. The linkage maps spanned 479 cM for HO with an average marker spacing of 9.6 cM and maximum gap size of 24.3 cM. For SS, the linkage maps spanned 478 cM, with an average marker spacing of 7.72 cM and maximum gap size of 22.3 cM (Table 1, Figure 1).

### Trichome density phenotypes

**Figure 2:**
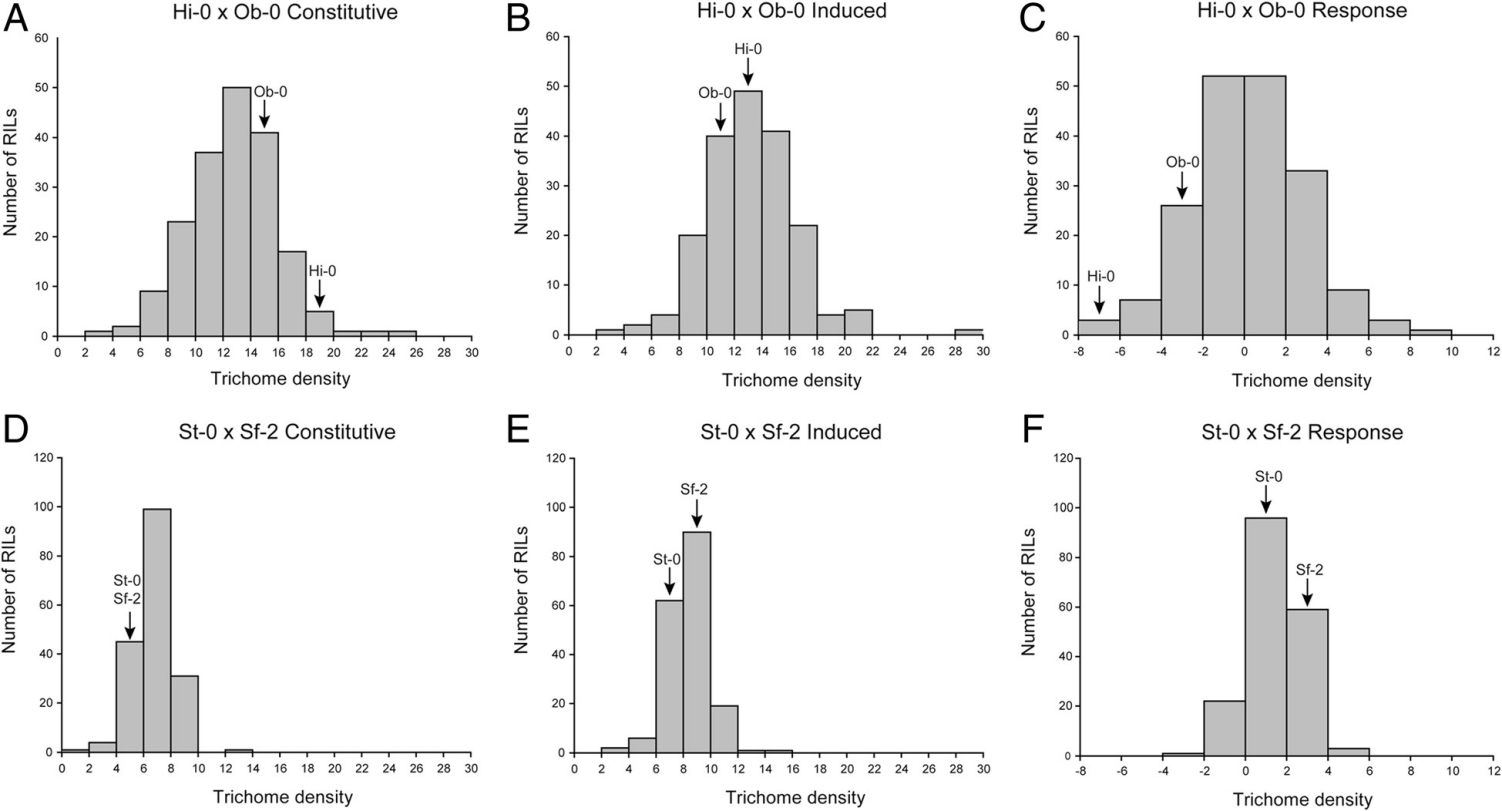
**Distribution of constitutive and induced trichome densities and response to damage for the Hi-0 x Ob-0 (A-C) and St-0 x Sf-2 (D-F) RILs and population parents.** Labelled arrows indicate the parental phenotypes’ positions in each distribution. Note that the Hi-0 and Ob-0 accessions and some proportion of RILs in both populations have negative responses to leaf damage.

Trichome density on the fifth rosette leaf was scored in the SS and HO populations in both control (constitutive) and damaged leaf (induced) environments. The difference in trichome density scores between the two environments was calculated for each RIL as a measure of the plants’ responses to leaf damage. In the HO phenotyping experiment the parental accessions showed constitutive trichome densities of 19.67 for Hi-0 and 15.67 for Ob-0 (Table 2, Figure 2). Surprisingly, both Hi-0 and Ob-0 had lower induced trichome densities than constitutive (13.5 and 12.0 respectively), resulting in a loss of 6.17 and 3.67 trichomes, respectively. The mean constitutive trichome density of the RILs was 13.25, increasing slightly to 13.72 when induced; this difference was weakly significant (p < 0.05) as determined by two-tailed paired T-test. The response to wounding of individual RILs in the HO population ranged from a decrease of 6.5 trichomes to an increase of 9.5 trichomes, with a mean increase of 0.48 trichomes. Transgressive segregation in the RILs was evident for all three trichome density phenotypes (Figure 2).

**Table 2:** **Parental and RIL mean trichome densities**^**1 **^**and standard error (SE), RIL range of trichome densities, and broad-sense heritability (H**^**2**^**)**

**Trait**	**Parental accession means ± SE**	**RIL mean ± SE**^**2**^	**RIL range**	**H**^**2**^
***Hi-0 x Ob-0***				
Constitutive	Hi-0 19.67 ± 2.60 Ob-0 15.67 ± 0.88	13.25* ± 1.41	6.0 – 25.5	0.74
Induced	Hi-0 13.50 ± 1.50 Ob-0 12.00 ± 1.00	13.72* ± 1.23	7.7 – 29.0	0.75
Response	Hi-0 -6.17 Ob-0 -3.67	0.48	−6.5 – 9.5	NA
***St-0 x Sf-2***				
Constitutive	St-0 6.00 ± 0.00 Sf-2 6.00 ± 0.58	7.06** ± 0.89	5.0 – 10.0	0.58
Induced	St-0 7.67 ± 0.88 Sf-2 9.33 ± 2.33	8.59** ± 0.85	5.7 – 12.7	0.62
Response	St-0 1.67 Sf-2 3.33	1.52	−3.3 – 4.7	NA

^1^The trichome density phenotype is described in detail in the Methods section.

^2^Average SE for individual RILs.

*Means are significantly different within a population at p < 0.05.

**Means are significantly different within a population at p < <0.001.

In the SS phenotyping experiment, the parental accessions St-0 and Sf-2 had identical constitutive trichome densities of 6.0 (Table 2, Figure 2). Both St-0 and Sf-2 increased trichome density when damaged to 7.67 and 9.33 respectively; this corresponds to a response to damage of a gain of 1.67 trichomes in St-0 and 3.33 trichomes in Sf-2. Mean constitutive trichome density of the RILs was 7.06, increasing to 8.59 when induced; this difference was highly significant (p < <0.001) as measured by two tailed paired T-test. The SS RILs also displayed transgressive segregation for all three phenotypes (Figure 2). Most SS RILs responded to damage by increasing trichome density, with a mean damage response of +1.52 trichomes but responses ranged from a decrease of 3.3 to an increase of 4.7 trichomes after wounding.

An ANOVA was used to calculate broad sense heritability (H^2^) of constitutive and induced trichome density in both populations. A strong genetic component underlies the observed variation in phenotypes. In the HO phenotyping experiment H^2^ was 0.74 for constitutive and 0.75 for induced and in the SS experiment, H^2^ was 0.58 for constitutive and 0.62 for induced. These values fall within the range of broad-sense heritabilities reported for trichome density and trichome number elsewhere [9-11,13]. As the “response” phenotype was calculated based on the mean trichome densities for each individual RIL in each environment, H^2^ could not be calculated.

### QTL for trichome density

**Figure 3:**
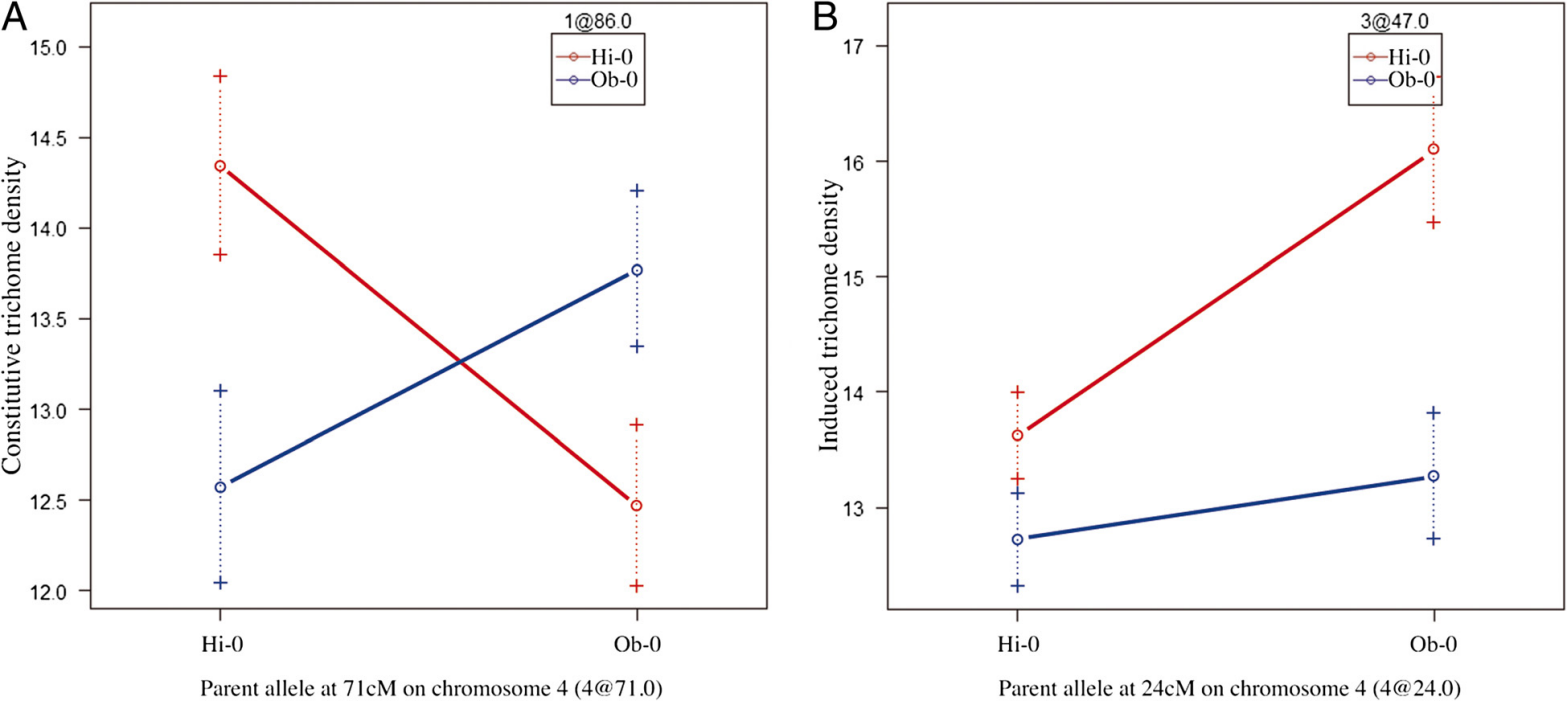
**Effectplots for epistatic interactions identified between pairs of QTL for the constitutive trichome density (A) and induced trichome density (B) phenotypes in the Hi-0 x Ob-0 mapping population.** Each panel shows the mean trichome density phenotype (y axis) for the four possible allele combinations found at two interacting loci. The parental allele at one QTL is indicated on the x axis and the parental allele at the interacting QTL is indicated by the color of the plot points and lines. Panel **A** shows a large interaction effect for constitutive trichome density between loci on chromosome 1 at 86 cM (HOC1, here labelled 1@86) and on chromosome 4 at 71 cM (HOC4, here labelled 4@71); the highest trichome density is achieved by genotypes where the alleles from the same parent co-occur. Panel **B** shows an interaction for induced trichome density between loci on chromosome 3 at 47 cM (HOD2; 3@47) and chromosome 4 at 24 cM (HOD3; 4@24). Here, the effect of the chromosome 4 locus appears to be masked by the Ob-0 allele on chromosome 3.

In the HO population QTL were mapped for both constitutive and induced trichome density, but no QTL were identified for the response phenotype. For constitutive trichome density, stepwiseQTL analysis produced two models with nearly identical pLOD scores. Due to their comparable pLOD values both models are presented in Table 3; the QTL results from Model 2, which appears to be the more comprehensive model, are presented in Figure 1. Model 1, with a pLOD of 7.32, identified four QTL, one each on chromosomes II and V, and two on chromosome IV, which together explained 34.26% of variation observed for this phenotype. Model 2, with a pLOD of 7.31, identified the same approximate QTL as Model 1 but included an additional QTL on chromosome I and an interaction between the chromosome I QTL and one of the two QTL identified on chromosome IV (Table 3, Figure 3); together, the QTL and interaction identified by Model 2 explained 54.25% of the observed phenotypic variation. The highest pLOD-scoring model for induced trichome density, with a pLOD of 6.05, identified individual QTL on chromosomes II, III and V and two QTL on chromosome IV with an interaction between the QTL on chromosome III and one on chromosome IV (Table 3, Figure 3). The QTL and interactions identified by this model explain 51.56% of variation observed for the leaf damage environment.

**Table 3:** Quantitative Trait Loci and epistatic interactions determined by stepwiseQTL analysis

**QTL**	**Chromosome**	**Position (cM)**	**Interval (cM)**	**Variation explained (%)**	**Allele mean trichomes**	**Candidate gene(s)**
***Hi-0 x Ob-0 Constitutive: Model 1; pLOD = 7.32***						
HOC2	2	48	40-62	6.46	H **14.3**; O 12.4	
HOC3	4	33	23-39	8.88	H 12.5; O **14.5**	
HOC4	4	82	67-84.8	5.61	H **13.7**; O 13.0	
HOC5	5	118	115-121	13.31	H **14.2**; O 11.8	
	TOTAL			34.26		
***Hi-0 x Ob-0 Constitutive: Model 2; pLOD = 7.31***						
HOC1	1	86	80-95	7.07	H 13.4; **O 14.2**	-
HOC2	2	48	42-67	5.04	**H 14.3**; O 12.4	ETC2/TCL1/TCL2^1^; TTG2; URM9
HOC3	4	34	26-38	11.27	H12.5; **O 14.5**	-
HOC4	4	71	65-84.8	11.83	**H 13.6**; O 13.1	-
HOC5	5	118	115-121	11.97	**H 14.2**; O 11.8	-
HOC1xHOC4^2^				7.07		
			TOTAL	54.25		
***Hi-0 x Ob-0 Induced; pLOD = 6.05***						
HOD1	2	52	38-62	5.92	**H 14.4**; O 13.1	ETC2/TCL1/TCL2;TTG2; URM9^1^
HOD2	3	47	43-58	10.76	**H 14.4**; O 13.0	GL1
HOD3	4	24	3-37	11	H 13.3; **O 14.7**	TT8
HOD4	4	79.5	77-81	12.72	**H 14.6**; O 13.1	-
HOD5	5	116	112-122	6	**H 14.3**; O 12.6	-
HOD2xHOD3^2^				5.16		
			TOTAL	51.56		
***St-0 x Sf-2 Constitutive; pLOD = 7.32***						
SSC1	1	107	102-109.7	7.84	**Sf 7.7**; St 6.7	GL2; At1g77670^1^; JAZ2
SSC2	2	61	52-68	7.15	Sf 6.6; **St 7.4**	URM9; TTG2^1^
SSC3	3	72	9-76	6.77	**Sf 7.4**; St 6.8	
SSC4	4	29	25-32	9.88	**Sf 7.3**; St 6.5	TT8
SSC5	5	11	5-13	9.84	Sf 6.6; **St 7.2**	
			TOTAL	41.48		
***St-0 x Sf-2 Induced; pLOD = 4.0***						
SSD1	1	102	87.92-109	12.34	**Sf 9.3**; St 8.3	RGL1; JAZ9^1^; JAZ2; GL2; At1g77670
SSD2	3	7.8	3-13	6.89	**Sf 8.9**; St 8.0	-
SSD3	5	4	0-108	6.99	Sf 8.2; **St 8.8**	-
			TOTAL	26.22		
***St-0 x Sf-2 Response; pLOD = 2.51***						
SSR1	1	64	58-71	11.98	**Sf 2.2**; St 1.2	-
			TOTAL	11.98		

^1^Denotes candidate gene in closest proximity to peak LOD score of the QTL.

^2^QTL x QTL interaction.

In the SS mapping population QTL were identified for all three traits. StepwiseQTL mapping for constitutive trichome density revealed a highest pLOD scoring model with five QTL, one on each of the five chromosomes, and no epistatic interactions; this model had a pLOD of 7.32, with QTL identified explaining 41.48% of observed variation for this trait (Table 3). The highest scoring model for induced trichome density, with a pLOD of 4.0, identified three QTL; one each on chromosomes I, III and V. These QTL together explain just 26.22% of the observed variation for this trait (Table 3). A single significant QTL underlying the variation in response of plants to leaf damage in this population was identified on chromosome I, explaining 11.98% of observed variation; of interest, this QTL does not overlap with significant QTL for constitutive or induced trichome density. No QTL were associated with the representative “cytoplasmic marker” in each population.

### Trait correlations

**Figure 4:**
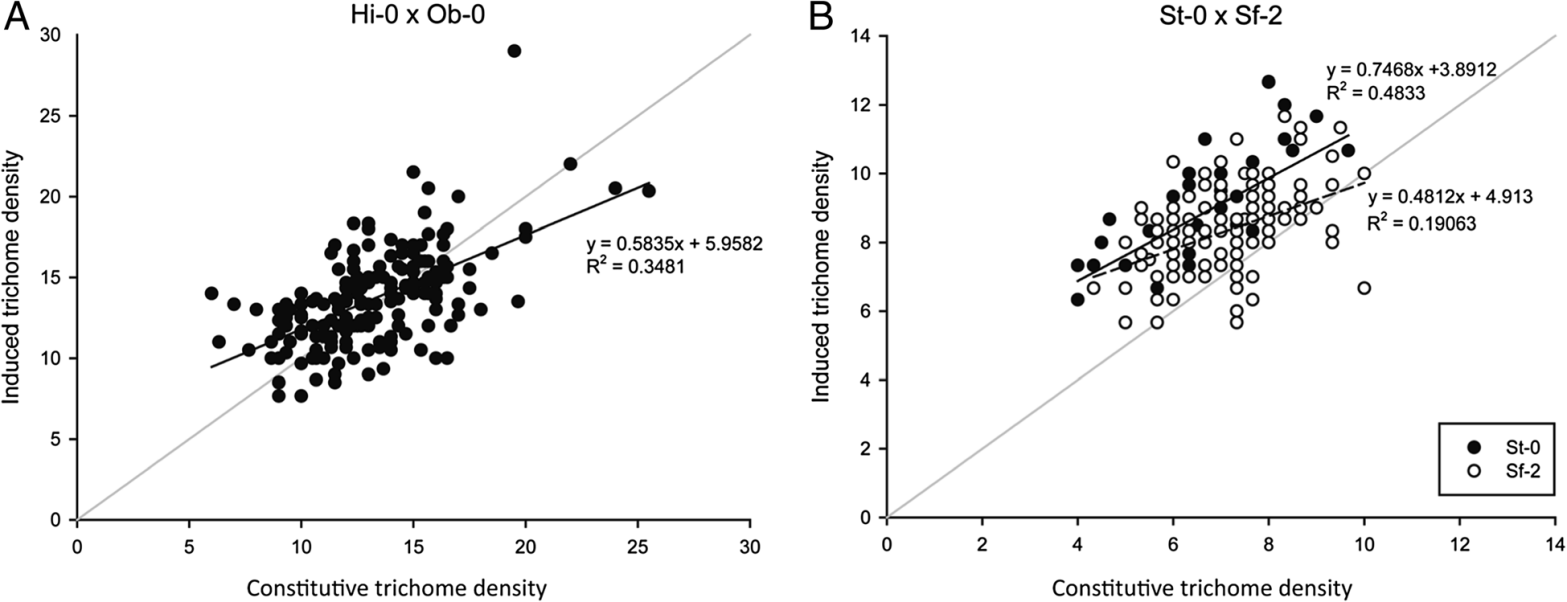
**Scatterplots for constitutive versus induced trichome density in the Hi-0 x Ob-0 (A) and St-0 x Sf-2 (B) RIL populations.** Because a QTL was mapped for the response phenotype in the SS population, those data were partitioned according to the allele carried by individual RILs at the marker nearest the response QTL. The gray diagonal indicates a slope of 1 on each graph; any point above the line therefore reflects RILs with a positive response to leaf damage (i.e., they increase trichome density) and any point that lies beneath the line are negative responders, which reduce trichome density in response to leaf damage. Separate regression equations and R^2^ values are shown for the two SSR1 genotypes in the SS population; the regression equation for the entire SS population is y = 0.543x + 4.7466 with an R^2^ = 0.2406.

To explore the relationship between constitutive and induced trichome density, within each mapping population, mean values for each RIL were plotted against one another in sigmaplot (Systat, Inc., Chicago, IL, USA). In each population, there was a positive correlation between constitutive trichome density and induced trichome density, but the slope of the regression line was considerably less than one (Figure 4). After applying a bias correction using an estimate of the reliability ratio ([37], chapter 1) as described by Holeski *et al.*[38], the uncorrected slopes shifted from 0.584 and 0.543 to bias-corrected slopes of 0.789 and 0.936 for HO and SS, respectively.

A single QTL, SSR1, was mapped for response in the SS population (Table 3, Figure 3). To examine the distribution of response phenotypes for different genotypes at SSR1, the phenotype data were partitioned into two sets: RILs with the St-0 genotype and RILs with the Sf-2 genotype at the marker nearest the QTL (msat1.42; Figure 4). Although each subset demonstrated a positive correlation, that for the Sf-2 RILs was ~2/3 that of the St-0 RILS for uncorrected and bias-corrected slopes alike. Intriguingly, the plot also shows that only RILs with the Sf-2 allele have zero or negative responses to leaf damage.

## Discussion

Plants deploy a dynamic range of defense strategies against herbivory, including plant hairs or trichomes. Because of the cost associated with trichome production and the variability in selection pressure put upon populations by herbivory, one might expect considerable standing variation for trichome density within or among populations, individual phenotypic plasticity for trichome density, or both. Numerous mapping studies have now demonstrated considerable genetic variation for constitutive trichome density in *A. thaliana*; however, although it also has been shown to be inducible, the genetic architecture of induced trichome density and response to damage have not been examined. Here, we utilized two new *A. thaliana* RIL mapping populations to investigate the genetic architecture of constitutive and inducible trichome density and the response to induction. Mapping in these populations identified new QTL for constitutive trichome density and identified the first for induced trichome density and response, as well as revealing an interesting qualitative shift in response to leaf damage.

### Trichome density phenotypes reveal plasticity and bidirectional variation for damage response

Heritabilities were relatively high for both traits in both populations (Table 2), indicating a strong genetic component to the observed variation, and were similar to heritabilities reported for trichome density previously [9-11,13]. Interestingly, heritability was slightly higher for induced trichome density than constitutive trichome density in both populations, perhaps suggesting that the damage treatment serves as a strong stimulus to the trichome initiation pathway, thereby reducing the relative effects of other environmental variables; however the difference is slight. While transgressive segregation (TS) is a common finding in both natural hybrid and mapping populations (reviewed in [39]), fairly dramatic TS was demonstrated for constitutive and induced trichome density in both populations described here (Table 2, Figure 2), particularly for the SS population, where the parents have identical constitutive trichome density phenotypes. TS may be a result of epistasis, overdominance, or parental accessions that each possess alleles with opposite effects [40]. Our results show that both epistasis and opposite effect alleles underlie trichome density variation in *A. thaliana* and provide an interesting case where the parental phenotypes belie genetic differentiation for a trait.

Trichome density distributions revealed intriguing and contrasting patterns in the two mapping populations. For example, both the Hi-0 and Ob-0 accessions were negative responders to induction, displaying lower trichome densities following damage (Table 2). In contrast, St-0 and Sf-2 had identical, and comparatively low, constitutive trichome densities and showed increased trichome density following damage. Likewise, the SS RILs had comparatively low mean constitutive trichome densities but showed a strong, significant increase of over 20% when damaged (induced). These observations would seem consistent with Optimal Defense Theory (reviewed by [41]), which predicts a negative correlation between the level of constitutive expression of a defensive trait and its capacity for induction. Similar results were reported in a recent mapping study of trichome production in *Mimulus guttatus*[38], with constitutive trichome density score negatively correlated with induction capacity. Here, this is further illustrated in plots of constitutive versus induced trichome density for RILs within each population (Figure 4). In the HO population, the slope of the regression line is positive but much less than one (Figure 4 and Results), indicating that as constitutive trichome density increases, induction capacity decreases. In the SS population, the uncorrected slope from linear regression of constitutive and induced trichome density is considerably less than one (Figure 4) but the bias-corrected slope is 0.936; this is further considered in the context of mapping results below. While plasticity for trichome density has been demonstrated in *A. thaliana* previously [12], the apparent relationship between constitutive and induced trichome density observed here has not.

### QTL mapping results identify epistatic interactions and a response (plasticity) locus

The pairs of parent accessions for the two populations differ considerably for all three phenotypes (Table 2, Figure 2). Hi-0 and Ob-0 are more different from one another for all traits than the St-0 and Sf-2 parents and yielded broader distributions for all traits in their RILs. Despite this, the total number of QTL mapped in each population was fairly similar (10 in HO and 9 in SS); however, the HO QTL explain more variation than the SS QTL for the constitutive and induced phenotypes.

#### Hi x Ob QTL

Of the QTL discovered in the HO population, four were mapped to similar positions for constitutive and induced trichome density and two were unique to one trait each (Table 3, Figure 1). In addition to the five main effect QTL identified in each environment, two strong epistatic interactions were identified (Figure 3). The interaction between HOC1 and HOC4 shows that constitutive trichome density is maximized when alleles from the same parent co-occur at these loci, while the interaction between HOD2 and HOD3 suggests a masking effect by the Ob-0 allele at HOD2 on the effect at HOD3 for induced trichome density. Interestingly, HOC1 was not identified as a main effect QTL through one-dimensional interval mapping (data not shown) and was instead only detected when considered as an interaction with HOC4, highlighting the importance of testing for epistasis to build more comprehensive models of genetic architecture. Epistatic interactions have been shown to underlie variation in a diverse range of traits in *A. thaliana* including fitness [42] and flowering time [43]. These interactions appear to be a potentially significant source of natural phenotypic variation, perhaps particularly where admixture introduces alleles into new genetic backgrounds.

#### St x Sf QTL

Despite the parent accessions having nearly identical constitutive trichome densities, a total of nine QTL were identified in the SS population (Table 3, Figure 1). Three QTL colocalized for constitutive and induced trichome density phenotypes (accounting for six of the nine QTL) and three were unique to a specific trait. A single QTL, SSR1, was mapped for response to wounding, which, interestingly, does not colocalize with significant QTL mapped for constitutive or induced trichome density. This contrasts with findings from work on another *A. thaliana* defensive trait that compared glucosinolate accumulation in control and methyl jasmonate treated plants, where all loci controlling phenotypic plasticity colocalized with QTL mapped in one of the two environments [44]. However, unique plasticity QTL have been mapped elsewhere, for example in barley [45] and rice [46]. As proposed under the gene regulation model of phenotypic plasticity [47], such QTL may represent regulatory loci, controlling plasticity by affecting expression of genes with a direct effect on phenotype.

### The SSR1 locus determines bidirectional variation for the response phenotype

Each mapping population possessed RILs with positive responses (increased trichome density) and RILs with negative responses (decreased trichome density) to leaf damage. The frequency of negative responders in the HO population was much greater than that in the SS population (Figure 4); this is not surprising as both parental accessions of this population are negative responders and both parents of the SS population are positive responders. Although no QTL for response was mapped in the HO population, one QTL (SSR1) was mapped in the SS population, indicating genetic variation for the trichome density response to leaf damage (plasticity). Interestingly, only RILs carrying the Sf-0 allele at SSR1 demonstrated zero or negative responses. Indeed, when the data are partitioned according to SSR1 allele, it is clear that RILs carrying the St-0 allele have, on average, a very different response to leaf damage across the constitutive trichome density distribution than those carrying the Sf-2 allele (uncorrected slopes of 0.75 and 0.48, respectively). When these slopes are bias-corrected (see Results), the responses demonstrate a qualitative difference in response trajectory. Specifically, for RILs carrying the St-0 allele, as constitutive trichome density increases, so too does the response to leaf damage (bias-corrected slope = 1.21) while RILs that possess the Sf-2 allele show the opposite response: as constitutive trichome density increases, the response to leaf damage decreases (bias-corrected slope = 0.867). This result suggests an allele-specific qualitative difference in response trajectory following leaf damage.

Despite the relatively high frequency of RILs that are negative responders, it is not immediately clear why plants would reduce trichome density in response to leaf damage. The result might suggest that for certain genotypes with high constitutive trichome density, making more trichomes isn’t necessarily a good strategy, therefore, plants may instead switch between defense strategies (e.g., producing more glucosinolates instead). The results described above identify allelic variation at a single locus that seems to determine the strategy employed (increasing versus decreasing trichome density as a response). Clearly, further work that focuses on the frequency and distribution of naturally occurring positive and negative responding genotypes within *A. thaliana*, identifying the genetic basis of the switch, and determining whether negative responders induce defense by other means would be of interest.

### QTL mapping confirms known and identifies novel loci for trichome density variation

Hi-0 x Ob-0 and St-0 × Sf-2 utilize parental accessions that previously have not been included in experimental mapping populations, and thus provide a new source of genetic variation from which to identify loci with a role in natural trait variation. Mapping trichome density in these populations uncovered loci that appear to overlap with QTL identified in other populations and loci that, to our knowledge, have not been mapped previously. Typically, mapped QTL span fairly large intervals containing many genes and, as such, different loci may underlie QTL mapped to similar positions in different populations or environments. Similarly, multiple contributing loci may be contained within a specific QTL interval. To provide a framework for identifying the genes that underlie QTL mapped here (and elsewhere), we estimated physical positions of LOD intervals from the physical positions of markers flanking the interval. Based on the extensive literature around the molecular genetic pathway for trichome initiation in *A. thaliana*, several strong candidate genes are identified (Additional file 3).

QTL mapped in multiple experimental populations may represent loci with key roles in the generation of trichome density variation within *A. thaliana* or simply high frequency polymorphisms within the species. Comparing our mapping results with previous studies, a number of loci appear to overlap with or fall near previously mapped QTL. In particular, three QTL, HOC2, HOD1, and SSC2, were mapped in close proximity to one another on chromosome II, overlapping with QTL mapped in this region previously in five different RIL and F_2_ mapping populations [9,11,14,26], and in a genome-wide association study of 191 *A. thaliana* natural accessions [13], explaining between 11 and 73% of the variation observed. The interval covered by HOC2/HOD2/SSC2 includes five obvious candidate genes: an array of trichome initiation repressor R3 MYB genes, *ETC2*, *TCL1*, and *TCL2*[18]; *TTG2*, a downstream target of the TTG1 pathway [48]; and *URM9*/*SAD2*, which links jasmonic acid signalling to trichome initiation via regulation of *GL3, GL1, TTG1* and *GL2*[24,49]. A lysine to glutamine mutation in *ETC2* has been suggested as the underlying quantitative trait nucleotide for this locus [26], although the effect of a combination of tightly linked polymorphic loci might explain why the percentage of variation reported for this locus is so variable among mapping populations and is so high in particular studies.

HOD2, mapped on chromosome III, appears to colocalize with QTL mapped in three RIL mapping populations including SS, Col x Ler [9] and Da(1)-12 x Ei-2 [15], a genome wide association mapping study [13], and an association mapping study of 94 accessions [10]. The TTG1 pathway MYB gene *GL1,* which associates with qualitative and quantitative variation in trichome density in natural accessions of *A. thaliana*[10,50], has been suggested as a candidate gene for this locus in previous studies [9][13]. HOD3, which partially overlaps with HOC3, SSC4 and a region previously mapped in L*er* x No-0 and Da(1)-12 x Ei-2 RILs [9,15], spans the physical position of the TTG1 pathway bHLH *TT8. TT8* does not yet have a demonstrated role in regulating trichome initiation on the leaf lamina but our mapping results, together with evidence for a role in trichome initiation on leaf margins and expression in the leaf lamina in response to jasmonic acid [19], suggests that such a role merits further study.

Several of the QTL mapped here neither overlap with nor fall in close proximity to previously identified loci, but instead appear to represent distinct, novel trichome density loci. Overlapping QTL on chromosome I, SSC1 and SSD1, are positioned near *GL2* and *At1G77670,* direct downstream targets of the TTG1 pathway [48,51]. SSD1 spans a larger interval than SSC1 that also includes, *RGL1*, a gibberellin response regulator with a role in trichome initiation [22], and *JAZ2* and *JAZ9*, jasmonic acid response regulators that interact with TTG1 pathway genes in yeast-2-hybrid assays [25]. HOC5/HOD5, which overlap on chromosome V, also appear to represent novel loci with no clear candidate genes. SSD2, on chromosome III, does not appear to have been mapped in experimental populations, although it may span *ELC*, a gene identified as a candidate trichome density locus in genome-wide association mapping [13]. Although candidate gene summaries such as this one cannot be comprehensive, the candidate gene approach to identifying the causal genes for trichome density variation in *A. thaliana* has proven particularly fruitful in the past [10,26,27].

## Conclusions

In this work, we have mapped QTL for trichome density in two new RIL populations of *A. thaliana*. The results show that, while there is some overlap between constitutive and induced trichome density QTL, roughly one-half of all QTL were mapped to just one trait. Importantly, we have identified QTL × QTL interactions and QTL for the response to damage (plasticity) that appear to be independent of constitutive and induced trichome density QTL. Drawing from a rich literature around epidermal cell fate and associated stress signaling pathways, a number of candidate genes are identified. Perhaps most interesting, our data also revealed qualitative variation for the response to leaf damage; i.e., some natural accessions and their RILs respond to damage by increasing trichome density and others respond by decreasing trichome density. Significantly, a QTL for this qualitative shift in response was identified, revealing a genetic basis for this novel pattern. Future efforts should focus on refining our understanding of the relationship between constitutive and induced trichome density, and identifying the polymorphisms that underlie the QTL mapped. Finally, the two new RIL populations have proven to be effective new tools for genetic mapping in *A. thaliana*. As the populations are segregating for many other traits, they should be of broad utility to the mapping community at large. Both seed stocks and genotypes are available from ABRC and NASC.

## Methods

### Plant materials

Based on preliminary screens of genetic and morphological variation in *A. thaliana* (e.g., rosette diameter, flowering time, and leaf serration), several pairs of natural accessions were selected to serve as progenitors for the development of new recombinant inbred line (RIL) mapping populations. Among those pairs were Hi-0 (CS6736)/Ob-0 (CS6816) (HO population) and St-0 (CS38906)/Sf-2 (CS6857) (SS population). Members of a pair were reciprocally crossed and the resulting F_1_s were confirmed to be cross progeny by genotyping several microsatellite loci and comparing the results with the parental accessions. F_1_s were allowed to self-pollinate and the seed was collected. Several hundred F_2_ seed were started in individual pots to establish individual RILs. Each F_2_ plant was followed through selfing and single seed descent for eight generations, ending in the bulk collection of seed from F_9_ plants. The bulked seed from 307 RILs of Hi-0 x Ob-0 and 261 RILs of St-0 x Sf-2 were deposited with the Arabidopsis Biological Resource Center (ABRC), where they were further bulked. Prior to genotyping, seed for each RIL population were acquired from the ABRC to assure that the genotypes match available seed lines. The progenitor accessions and 188 randomly chosen RILs from each population were used in the present study.

### Marker screening

The parent accessions of both populations, Hi-0, Ob-0, St-0 and Sf-2, were grown under standard growth room conditions of 24°C, 16:8 hours light:dark. Genomic DNAs were extracted from fresh tissue obtained from young rosette leaves using a modified CTAB extraction protocol [52]. These DNAs were used to screen for microsatellite markers polymorphic between parental accessions (details below) initially using primers from the French National Institute for Agricultural Research (INRA) Microsatellite Database (http://www.inra.fr/internet/Produits/vast/msat.php). Additional primers were designed in Primer3 based on flanking sequences from microsatellite repeats identified from the Eukaryotic Microsatellite Database (http://www.veenuash.info/) or by our lab using the Col-0 reference genome sequence (Arabidopsis Genome Initiative 2000). In excess of 150 microsatellite markers were screened to identify markers for linkage map development in the HO and SS populations; of these, many were monomorphic or amplified alleles in only one or neither parent.

An M13 primer-tailing scheme polymerase chain reaction (PCR) was used for genotyping. For the initial screen for polymorphic loci, markers were amplified in 10 μL PCR containing 1X NEB Thermopol buffer (New England Biolabs, Ipswich, MA, USA), 2 μmol dNTPs, 0.2 μmol M13-tailed marker-specific forward primer, 4.5 μmol reverse primer, 4.5 μmol FAM-labelled M13 primer, 0.5U NEB Taq polymerase, and ~50 ng genomic DNA under the following cycling conditions: 95°C for three minutes, followed by 30 cycles of 95°C for 30 s, 52°C for 40 s, and 72°C for 40 s, and a final extension of 20 minutes at 72°C. One microliter of PCR product was then combined with 9 μL of a HiDi (Applied Biosystems) and CASS size standard [53] mix. Allele size was determined by capillary separation of fluorescently labelled PCR products and CASS size standard on an ABI3730 Genetic Analyzer (Applied Biosystems, Carlsbad, CA, USA) at the Massey Genome Service; parental allele sizes were called in genemapper v3.7 (Applied Biosystems). Consistently amplifiable markers polymorphic between the parents of a population were chosen for RIL population genotyping.

### RIL genotyping and linkage map construction

A set of 188 RILs plus parents were screened in each mapping population. RIL genotyping PCRs were tailed with M13 primers labelled with one of three fluorescent dyes, FAM, VIC or NED, and PCRs carried out as described above. Three markers (each with a different fluorescent label) from the same individual were pooled for capillary separation. Markers selected for a pooling group had non-overlapping allele sizes. After individual amplification reactions, markers were pooled together in a ratio dependent on the strength of amplification of each marker in parental screens. One microliter of the pooled markers was combined with 9 μL HiDi/CASS prior to capillary separation. Results were assessed and allele calls made using genemapper v3.7 (Applied Biosystems). When homozygosity was not absolutely clear individuals were conservatively scored as heterozygotes, thereby eliminating those genotypes from further analyses.

Ultimately, the HO population was genotyped at 55 loci and the SS population at 67 loci, with 32 markers common to both populations. Markers used for each population are listed in Additional file 1. Of the 188 RILs screened in each population, seven individuals were removed from HO and seven individuals from SS before linkage map construction and QTL mapping due to ambiguity of allele calls across a high proportion of markers. Linkage maps for both populations were constructed in joinmap4[35] using maximum likelihood.

### Trichome density phenotyping

The HO and SS populations were each phenotyped in separate experiments. To score trichome density phenotypes, six replicates of each RIL and their respective parents were planted in seed raising mix in 72 cell flats in a fully randomized design; approximately 5–10 seeds were sown in each cell. Seeds were stratified for eleven days at 4°C in the dark to synchronise germination times and then moved to a growth chamber at 24°C under sixteen hours light for 25 days post-germination. Plants were thinned to three per cell at four days post germination, and to a single plant seven days post germination. To map “induced trichome density”, three of the six replicates of each RIL were randomly selected to have leaves damaged (a small pilot study indicated that phenotypes were stable enough to use three replicates). Plants in the leaf damage experiment were subjected to pinching of one cotyledon with serrated forceps at four days post-germination, and first and third leaves shortly after emergence at seven and ten days post-germination, respectively. The remaining three (nonpinched) replicates of each RIL and parent accession were used to measure “constitutive trichome density”.

Trichome density was scored at 25 days post-germination. At this stage of development the fifth true rosette leaf was fully expanded and not yet senescent. Trichomes were counted on the fifth leaf in a 17 mm^2^ area midway along the length of the leaf blade between the midrib and leaf margin using a dissecting microscope at 25x magnification. Broad sense heritability (H^2^) was calculated independently for damaged and undamaged treatments of each population using mean squares from an ANOVA with RILs as groups.

### Trait correlation and QTL mapping

For each population, mean trichome density was calculated for each RIL in both treatments described above in excel (Microsoft, Inc.); henceforth these phenotypes are referred to as “constitutive trichome density” and “induced trichome density”. In addition, the plasticity of trichome density in response to damage was calculated for each RIL as the difference between mean constitutive and mean induced trichome densities; this is the “response” phenotype. The calculated RIL means for the three traits were used as phenotype values in QTL mapping and for trait correlation analyses.

To explore the relationship between trichome density traits, within each mapping population, constitutive and induced trichome density mean values for each RIL were plotted against one another in sigmaplot (Systat, Inc., Chicago, IL, USA). Because slope is potentially biased downward in linear regression due to estimate error in the predictor variable, we have applied a bias correction using an estimate of the reliability ratio ([37], chapter 1) as described by Holeski *et al.*[38]; essentially each slope is multiplied by the appropriate heritability (reliability ratio).

The five linkage groups for each population and a hypothetical “cytoplasmic marker” were used for QTL mapping. As each RIL population was generated by reciprocally crossing the parents, the origin of the cytoplasmic genomes (which are maternally inherited) is known for each RIL. As such, a hypothetical “cytoplasmic marker” was added to the dataset; alleles for this marker were assigned according to the maternal parent of each RIL. QTL analyses were carried out on RIL means using the R/qtl package [36]. Initially, variation for each trait was mapped using the “em”, “haley-knott”, and “multiple imputation” (256 replicates) methods in a 1D scan (scanone) and followed by a 2D scan (scantwo) for interacting QTLs using the same methods. The results for each model were compared and found to be qualitatively similar for any one phenotype for any one population. The number of QTL for each trait was estimated based on these initial analyses and used to assign a liberal maximum number of QTL in “stepwiseqtl” analyses (max.qtl). 2D permutation tests of 1000 reps were run using haley-knott regression; these results were used to derive the penalties used in stepwiseqtl analyses. The stepwiseqtl analysis steps through main effect QTL models, while refining positions, and searching for interacting QTL at every step; the scan.pairs option was selected. The models are progressively more inclusive, building from one main effect QTL up to the maximum (as estimated from initial 1D and 2D scans) and then stepping back down to one main effect QTL. Model outputs are compared by a penalized LOD (pLOD) score, which are calculated at each step and the model with the highest pLOD score is taken as the best fit to the data. The pLOD approach allows one to directly compare the fit of models of different size (different numbers of QTL). As the penalties for adding epistatic interactions are quite heavy, it was rare that best fit models included any such interactions; however, the data often showed strong evidence of interacting QTL in initial 2D scans. Ninety-five percent Bayes credible intervals for each QTL were calculated in R/qtl using the Bayesint function.

The physical positions of maximum LOD peaks and intervals were estimated from the physical positions of flanking markers in the Col-0 genome sequence, assuming a correlation between physical marker positions in the RILs with their physical positions on the Col-0 reference genome and a roughly linear relationship between physical and linkage positions. Candidate genes for trichome density QTL were identified from the extensive literature describing trichome initiation, trichome initiation signalling, and downstream regulatory targets. Map positions of the candidate genes identified as falling within a given 95% Bayes critical interval for a QTL were estimated using the map positions of markers with physical positions flanking the gene, based on assumptions outlined above.

### Availability of supporting data

The data sets supporting the results in this article are available from Dryad: doi:10.5061/dryad.2bs8g.

## Competing interests

The authors declare that they have no competing interests.

## Authors’ contributions

VS and AL received grant support for the project. VS, RB, and AL designed all experiments. VS generated the RIL populations. RB phenotyped and genotyped the populations. VS and RB constructed linkage maps, carried out QTL mapping, and performed remaining data analyses. RB and VS prepared the manuscript. All authors reviewed and approved the final manuscript.

## Supplementary Material

Additional file 1 Details for microsatellite markers used to generate linkage maps for Hi-0 x Ob-0 and St-0 x Sf-2 mapping populations. Click here for file

Additional file 2 **Genotype frequencies for each marker in each population.** Segregation distortion is present in both populations but is considerably stronger in the St-0 x Sf-2 population. Click here for file

Additional file 3 Candidate genes and associated AGI numbers for trichome density QTL. Click here for file
