# Supplementary material for: The genetic architecture of constitutive and induced trichome density in two new recombinant inbred line populations of Arabidopsis thaliana: phenotypic plasticity, epistasis, and bidirectional leaf damage response
Source: BMC Plant Biol. 2014 May 5;14:119. doi: 10.1186/1471-2229-14-119 (PMC4108038; doi:10.1186/1471-2229-14-119)
Supplement: Additional file 1 — Details for microsatellite markers used to generate linkage maps for Hi-0 x Ob-0 and St-0 x Sf-2 mapping populations. [file 1471-2229-14-119-S1.pdf]

Additional File 1. Details for microsatellite markers used to generate linkage maps for Hi-0 x Ob-0 and St-0 x Sf-2 RILs

Marker	Chromosome	Physical Position (bp)	HO Map position (cM)	HO Significant SD	SS Map position (cM)	SS Significant SD	Source	Forward primer	Reverse primer
**M100008**	1	8639	0	**			VAST	CCACGCATCTGTGTTCACTC	GCAAAAGTGGGCCTAACAAA
**F19P19**	1	1157727	2.598	***	0†	*****	VAST	CCACGTAGGTCAAGAAGAAGAAG	TGTCTGCTGCGATAGAGAGAG
**T1G11**	1	1243352			0.424†	*****	VAST	GAAGACAAAGCTCTGCAGTAATG	AATTGCATAAGGCACTTGAAAG
**F21M12**	1	3212191	6.784	**	5.105	*****	VAST	GGCTTTCTCGAAATCTGTCC	TTACTTTTTGCCTCTTGTCATTG
**ICE10**	1	4015381	10.47	**			VAST	AACATCCACAAGTTTCTAAAACAATC	GACTCTTATGGGTAAGCTCCTTG
**MS1-2538**	1	6165662			13.956	*****	VAST	TGGGTTTTTAATGGCGTTTG	CCGGCGACTTATGCTTACC
**MSAT1.10**	1	7296649	22.834	-			VAST	ATGGTGAGATACTGAGATTAT	CGAGAAGGTCTAAAGGTA
**MSAT1.3**	1	8322175	29.082	*****	28.392	****	VAST	GGAACTGTTGTCTGGGTAAG	CGATTGCACTAAAAGCTCTC
**CIW12**	1	9621344			36.254	*****	VAST	AGGTTTTATTGCTTTTCACA	CTTTCAAAAGCACATCACA
**VMS1.10**	1	10	40.157	-			This paper	ACCAACTCGGATTCGCCTCG	TTTGCTGGCGATATGAGTCACTTG
**MS1-5284**	1	12511232	54.917	-			EuMicrosat	CCTGAAAACCCCGTTAAAAG	CGGGACCTTTGTGTGATATTC
**MSAT1.4**	1	14160180	63.02†	-			VAST	CTAAACTAGAACCAGGGGTAA	ACAAAAATCGTGGTGATAATA
**T27K12**	1	15926702	64.389†	-	54.593	*****	VAST	GGAGGCTATACGAATCTTGACA	GGACAACGTCTCAAACGGTT
**MSAT1.42**	1	18153083	74.759	-	65.695	*****	VAST	TAACTAAAATTGCACCC	ACAAATAAAATTGAATATCAG
**NGA128**	1	20633251	87.511	-	76.226	*****	VAST	GGTCTGTTGATGTCGTAAGTCG	ATCTTGAAACCTTTAGGGAGGG
**F5I14**	1	24374008			87.92	*****	VAST	CTGCCTGAAATTGTCGAAAC	GGCATCACAGTTCTGATTCC
**MSAT1.12**	1	25686587			93.066	*****	VAST	TTAGAGATTCGCCAACCTC	CGTGTGCCCAACCA
**MSAT1.13**	1	25827433			96.928	*****	VAST	CAACCACCAGGCTC	GTCAAACCAGTTCAATCA
**MSAT1.1**	1	26398711	106.553	-	98.67	*****	VAST	ATACGATAAGATTTATTAGCA	CCCATGCTCTTTTTGTGAAA
**F22K20**	1	28895040	116.332	-	104.549	*****	VAST	TTTTTGGTGAGATTTTAAGCCC	ATATCTCCATCGCTGCAACC
**MSAT1.5**	1	29016886			104.557	*****	VAST	GCATCGCTCTTAAACAACCAT	CGTTGCAAAACCGTATCAGAA
**F5I6M**	1	30192367			109.661	*****	VAST	GACCCTAGAGGCAAACCAAATG	AGTGAGTAAATGGTGGGGAATCG
**MSAT2.5**	2	208178			0	*	VAST	TGAGAGGGACAGATAGGAA	ATCAAAAGGGATACTGACAA
**MSAT200897**	2	897197	0	-	10.632	**	VAST	AAAAATTTTAACCCATGTTGTAGCA	AGAAGTCGGTGGATGAGCAC
**MSAT2.38**	2	2457014			19.183†	****	VAST	TGTAACGCTAATTTAATTGG	CGCTCTTTCGCTCTG
**MSAT2.18**	2	2799644	8.528	-	19.819†	****	VAST	TAGTCTCTTTTGGTGCGCATA	AGCCTCTCCAAGCTTAGGTCT
**IND628**	2	6280231			23.184†	****	VAST	AATTTAAGAAAGAACACAATTTTGAGA	CCATTTCAGCTCTTTACACTTCTACA
**MSAT2.28**	2	6409845			24.096†	-	VAST	AATAGAAATGGAGTTCGACG	TGAACTTGTTGTGAGCTTTG
**MSAT2.11**	2	8227826	24.945	*			VAST	GATTTAAAAGTCCGACCTA	CCAAAGAGTTGTGCAA
**PLS7**	2	9813750	29.735	-			VAST	GATGAATCTTCTCGTCCAAAAT	GACAAACTAAACAACATCCTTCTT

**MSAT2.31**	2	10778033	33.798	-			VAST	GCTCCTCTTTGCCGCTAG	GCGATTTCATCTTGTGCATC
**MSAT2.41**	2	11095452			42.648	**	VAST	GACTGTTTCATCGGATCCAT	ACAAACCATTGTTGGTCGTG
**MSAT2.7**	2	13192607	47.843	-			VAST	CTCAAATCAAGAACGCTGAC	CCCGATATAGACAACGACAA
**MSAT2.4**	2	13831870			54.808	*****	VAST	TGGGTTTTTGTGGGTC	GTATTATTGTGCTGCCTTTT
**IND216199**	2	16199330	55.207	-			VAST	TGGCTCATTTCACTCACAGG	GGCTATCTATGGCCGTTATGA
**MSAT2.22**	2	19632943	69.455	-	75.988	**	VAST	CGATCCAATCGGTCTCTCT	TGGTAACATCCCGAACTTC
**MSAT3.99**	3	1514825	0	-	0	*****	VAST	CCAACAAGAACAAGACATT	AGTGAAATCTGAAGCGATA
**ATHCHIB2**	3	3963391	11.193	-	3.039	*****	VAST	GGATCCAAGTGCTCATATATAC	CTTTCGTTTCTAAATATGAGAAGC
**NT204**	3	5570082	17.117	-			VAST	TGGAAGCTCTAGAAACGATCG	ACCACCTAAACCGAGAATTGG
**MSAT305754**	3	5754608			7.814	*****	VAST	AGAAGAAGCGCACATGTTTTT	AAAATTGTACGTTTGATTGTTGAA
**MSAT3.4**	3	7672410	23.793	-	17.718	**	VAST	TGTAACATTCGCATTTTATG	CCTCTTCGGTCTATATGACT
**MSAT3.6**	3	8467190			25.118	***	VAST	CTACTTTACGCCTTTTATTG	TGAATTGTCCCGAGAC
**MSAT3.19**	3	8808167	36.21	-	29.328	-	VAST	TAATTCGATCCAATTGACAT	TGGCTTGGCACAAAC
**MSAT3.2**	3	9055511			30.85	-	VAST	AAGGTACGGCGGTGGATATTG	CGGGGATTTCTTCTTCCTGTG
**MSAT3.117**	3	9860185	41.648	-	38.296	**	VAST	CCCGTTTAGAAATATGTGT	CGTCATACCATTTTATCAG
**MSAT3.8**	3	11002949	49.586	-			VAST	ATGTTAAAAACCCGTGTTGG	TTTAACCTTATCCGGGAAAG
**MSAT3.31**	3	11037360			48.772	**	VAST	CTCTTTTTCTTATGCCAA	AGAGTGGGTTGATACCAT
**MSAT3.32**	3	11208231			52.728	****	VAST	GCACTTGCAGCTTAACTT	CGTGACTGTCAAACCG
**MS3-5962**	3	15392985	53.817	-			EuMicrosat	CACGTGCGAACGACTACAAC	GCTCCAAAACGGAACCCTAC
**VMS3.17**	3	17	63.063	-			This paper	GCCGGGCTACAAGCCCAGAAAAG	ACCGGTGAGGAATGGCCAGC
**MSAT3.65**	3	19835420	86.335	-	74.988	-	VAST	CCAACTCTTCTTTCAAACA	TCTCTTAGCAAACGAATGTC
**CDC2BG**	3	20070710			77.153	*	VAST	GGGAAAAACGAAGTGACGTG	ATTGAACTGTGTTGGTTTCTGG
**MSAT3.13**	3	21377089			84.888	-	VAST	TTGTGTGTTTGCGATC	CATATCCGTTTTTATGTTTT
**NGA6**	3	23042025			90.284	**	VAST	TGGATTTCTTCCTCTCTTCAC	ATGGAGAAGCTTACACTGATC
**MSAT3.70**	3	23449179			91.296	***	VAST	CACCCATAGACACACAACC	TCAAAACCTCCAACCATAC
**MSAT4.41**	4	42208	0	*****	0†	-	VAST	CCTACACTCATCAGATTCAG	TAATTAGGGTTTGATCATTC
**MSAT4.39**	4	89498			0.739†	-	VAST	GTTATCACATTAAAATCACC	CCAATTGTAATATATGAACA
**MSAT4.43**	4	2575724	11.23	*****	10.854	-	VAST	GCATTGGACCATAAGGG	TCAAGACGGTTTCAAGTTG
**ATHDET1**	4	6346349	19.741	*****	29.233	*****	VAST	TTCAAACACCAATATCAGGCC	GGTGAAAATGGAGGAGACGA
**MSAT4.35**	4	7549254	28.94	****	33.361	*****	VAST	CCCATGTCTCCGATGA	GGCGTTTAATTTGCATTCT
**MSAT4.15**	4	9362588	41.419	-	40.877	****	VAST	TTTCTTGTCTTTCCCCTGAA	GACGAAGAAGGAGACGAAAA
**CIW7**	4	11524362	55.634	*	56.022	**	VAST	AATTTGGAGATTAGCTGGAAT	CCATGTTGATGATAAGCACAA
**MS4-5228**	4	13421331			64.347	-	EuMicrosat	GCTCCTCGAGAAAAAGAACG	TCTCACATCACGGGACAATC
**MSAT4.14**	4	15212515	72.547	**	70.668	**	VAST	GACCGTTTCTAGTGCTCACA	ACGGAATAAGCGGAGGA
**MSAT4.12**	4	15960078	73.708	*			VAST	AAAGGAAGAAGAAGACTGTT	AGAAGAAGAAGCGAGATT

**MSAT4.68**	4	17261718	79.464	**	80.69	-	VAST	GGTCTCTTTTTCCCTTAAC	CATTGTCCATCATCAGAAC
**MSAT4.38**	4	18412248	84.821	*	88.353	-	VAST	GCCTTATAGTACACCCAAA	CCACTCCACTCTCGAA
**MSAT4.31**	4	18573159			88.848	-	VAST	AGGGATATGGATTGAGA	GCCGTATAACTATTGGTT
**MSAT500027**	5	27394			0	*****	VAST	AAAAGGAAGACAAAGCTTGACG	AACCCCACATGGCCTTATTT
**ATHCTR1A**	5	979763	0	**			VAST	TATCAACAGAAACGCACCGAG	CCACTTGTTTCTCTCTCTAG
**NGA225**	5	1507104	1.475	***	8.504	*****	VAST	GAAATCCAAATCCCAGAGAGG	TCTCCCCACTAGTTTTGTGTCC
**NGA151**	5	4669932			16.457	*****	VAST	GTTTTGGGAAGTTTTGCTGG	CAGTCTAAAAGCGAGAGTATGATG
**NGA106**	5	5397351	12.377	-			VAST	GTTATGGAGTTTCTAGGGCACG	TGCCCCATTTTGTTCTTCTC
**MSAT5.14**	5	7498509	25.871	-	23.619	*****	VAST	AACAACCCTATCTTCTTCTG	TGTGACCCCTTACTCAATA
**NGA139**	5	8428136	31.946	-	27.093	*****	VAST	GGTTTCGTTTCACTATCCAGG	AGAGCTACCAGATCCGATGG
**MS5-4717**	5	10960119	54.19†	**			EuMicrosat	GCCAATCCGAGTAAATCGTG	GGTGGACCGTTCCATTTTG
**MSAT512110**	5	12109959	54.759†	**			VAST	GATCTGATATACCTCGATTTTC	TTTAATAAGTCTTTTCTCTACGC
**MSAT5.1**	5	13924203			31.696	*****	VAST	TCTAGCTTTATCCTTCTTTGA	GCCTATTCCGAGATTCA
**MSAT5.22**	5	13961058	60.913†	-	39.808	*****	VAST	AGAACAAGTTAGGTGGCT	GGGACAAGAATGGAGT
**MSAT5.59**	5	15461361			51.898	*****	VAST	CATAAGTCAGTTGATTCCA	GCTAAAACTAGAGCGAGAG
**MSAT5.7**	5	17071433	79.241	*****			VAST	GCTTGTACATTACCGGACA	CCAAGCGTACTCGATCA
**MSAT5.8**	5	17163228			57.879	*****	VAST	GAAACATTTCATGGGCTTT	GAGAGCAGAGGCAAGTCA
**MSAT518662**	5	18662765	86.687	*****	64.629	*****	VAST	GCTCCAAAACCCAACAGGTA	TGGCCTCAAAGCTACCCTTA
**MSAT520037**	5	20037472			71.238	*****	VAST	CTTTCGGTGGATAAATCATGTTA	CGCATACAAATTTTCTGCTATGT
**JV6566**	5	22027830			82.103	*****	VAST	TTTCTTTCACGGTTTCTAACTTTT	TCATCTTCCCTTTAATTCTGATGA
**JV6162**	5	22527623	110.994	*****			VAST	CGCTTTCCTTGTGTCATTCC	AAATGCAAATATTGATGTGTGAAA
**ICE4**	5	23837141	115.024	*****			VAST	CACGAGGAATCTGGCATGGTCG	AGCGATTGCAAGCGGCTCAAG
**JV7576**	5	23879358			93.282	**	VAST	CACAATCAGAGGGGGTTGAT	AAATTTTGGGGGAAATGAAA
**MSAT5.19**	5	25924795	122.18	*****	98.609	-	VAST	AACGCATTTGCTGTTTCCCA	ATGGTTATCTCATCTGGTCT
**K9I9**	5	26983961			112.624	-	VAST	GATTCAGAGTTACGGTAACAG	CTATTGACAGTAAAGAGTCG

SD: Segregation distortion; Significance levels - >0.1 *<0.1 **<0.05 ***<0.01 ****<0.005 *****<0.001

† Indicates markers constrained to match physical positions

VAST database http://www.inra.fr/internet/Produits/vast/msat.php

Eumicrosat database http://www.veenuash.info/
