# Supplementary figures and images for: The genetic architecture of constitutive and induced trichome density in two new recombinant inbred line populations of Arabidopsis thaliana: phenotypic plasticity, epistasis, and bidirectional leaf damage response
Source: BMC Plant Biol. 2014 May 5;14:119. doi: 10.1186/1471-2229-14-119 (PMC4108038; doi:10.1186/1471-2229-14-119)

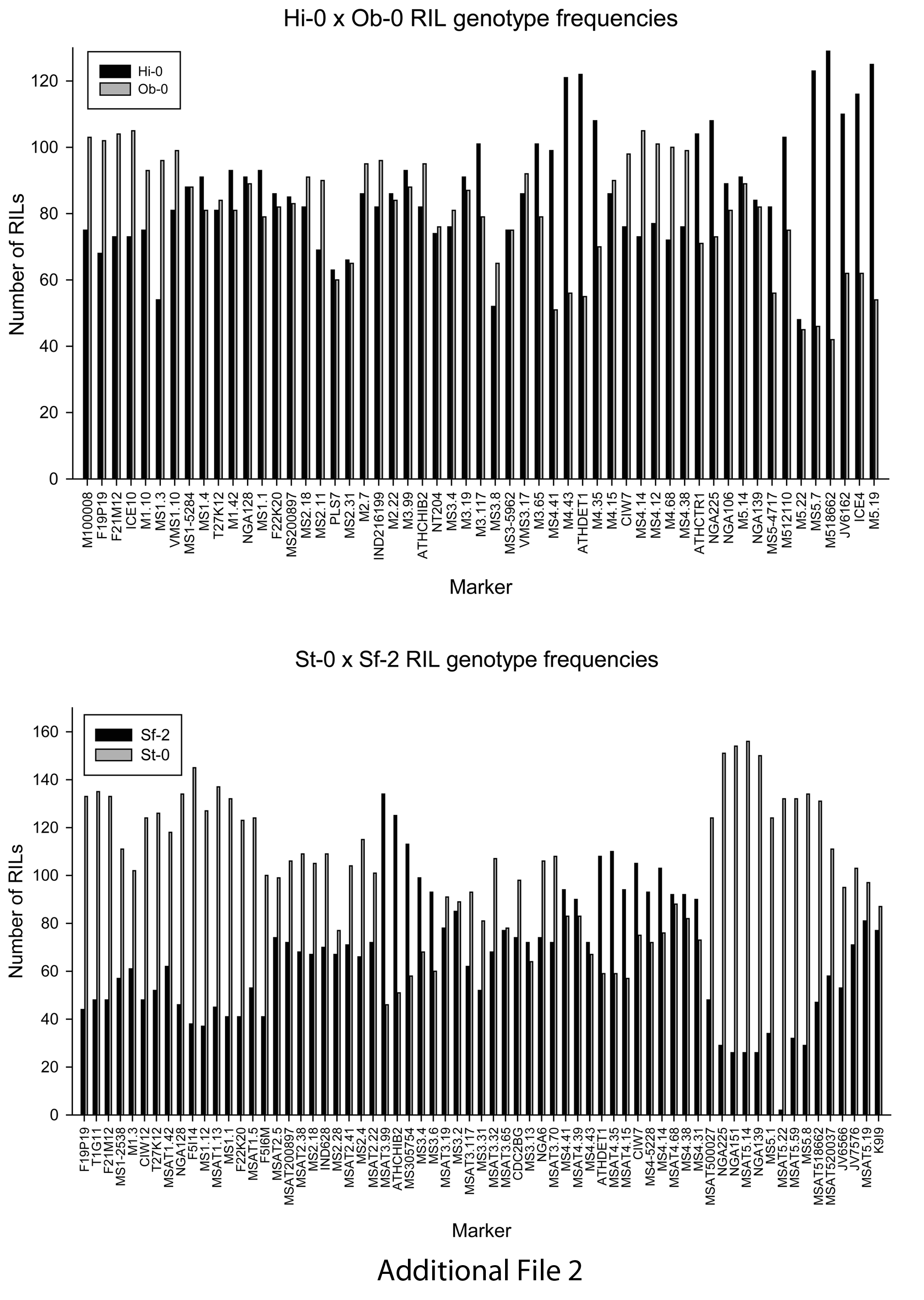

Supplement: Additional file 2 — Genotype frequencies for each marker in each population. Segregation distortion is present in both populations but is considerably stronger in the St-0 x Sf-2 population. [file 1471-2229-14-119-S2.tiff]
