# Supplementary material for: The genetic architecture of constitutive and induced trichome density in two new recombinant inbred line populations of Arabidopsis thaliana: phenotypic plasticity, epistasis, and bidirectional leaf damage response
Source: BMC Plant Biol. 2014 May 5;14:119. doi: 10.1186/1471-2229-14-119 (PMC4108038; doi:10.1186/1471-2229-14-119)
Supplement: Additional file 3 — Candidate genes and associated AGI numbers for trichome density QTL. [file 1471-2229-14-119-S3.pdf]

Additional File 3. Candidate genes and associated AGI numbers for trichome density QTL.

QTL	Candidate gene	AGI Number
**HOC2, HOD1**	*ETC2*	At2G30420
**HOC2, HOD1**	*TCL1*	At2G30432
**HOC2, HOD1**	*TCL2*	At2G30424
**HOC2, HOD1, SSC2**	*TTG2*	At2G37260
**HOC2, HOD1, SSC2**	*URM9* (*SAD2*)	At2G31660
**HOD2**	*GL1*	At3G27920
**HOD3, SSC4**	*TT8*	At4G09820
**SSC1, SSD1**	*GL2*	At1G79840
**SSC1, SSD1**	*At1G77670*	At1G77670
**SSC1, SSD1**	*JAZ2*	AT1G74950
**SSD1**	*RGL1*	AT1G66350
**SSD1**	*JAZ9*	AT1G70700
